# Effect of Public Empathy with Infection-Control Guidelines on Infection-Prevention Attitudes and Behaviors: Based on the Case of COVID-19

**DOI:** 10.3390/ijerph182413408

**Published:** 2021-12-20

**Authors:** Eugene Song, Jae-Eun Lee, Seola Kwon

**Affiliations:** 1 Department of Consumer Science, Chungbuk National University, Cheongju 28644, Korea; eugenesong@chungbuk.ac.kr; 2 Department of Public Administration, Chungbuk National University, Chungbuk 28644, Korea; jeunlee@chungbuk.ac.kr; 3 National Crisisonomy Institute, Chungbuk National University, Chungbuk 28644, Korea

**Keywords:** public empathy, infection, attitude, behavior, COVID-19

## Abstract

Since the outbreak of the novel coronavirus disease (COVID-19), the government has provided infection-control guidelines to prevent the spread of the virus. The authors of this study examined the structure (causal relationship) of factors that influence public behavior toward COVID-19 and verified the effect of public empathy with infection-control guidelines in each structure. Data were collected using a self-administered questionnaire survey from 211 Korean adults. The results showed that (1) the perceived susceptibility and severity of economic damage had a positive effect on infection-prevention attitudes and infection-prevention attitudes had a positive effect on infection-prevention behaviors; (2) the perceived severity of economic damage had a positive effect on infection-prevention attitudes; and (3) public empathy with infection-control guidelines positively moderated the effect of the perceived severity of economic damage on infection-prevention behaviors and that of perceived susceptibility on infection-prevention attitudes. Accordingly, the authors of this study present the following three suggestions to prevent the spread of an infectious disease: engage in risk communication focused on a potential virus infection and cooperation, make multifaceted efforts to increase public empathy with infection-control guidelines, and implement measures to alleviate or reduce economic damage to the public in a viral pandemic.

## 1. Introduction

Severe acute respiratory syndrome coronavirus 2 (SARS-CoV-2) was first observed in January 2020 in Wuhan, Hubei Province, China, where pneumonia was prevalent [1,2]. According to Johns Hopkins CSSE COVID-19 daily reports, as of 31 August 2021, there were 217,089,516 confirmed cases and 4,509,857 deaths, with a mortality rate of 2.1% [3]. The National Health Service (NHS) in the UK published COVID-19 guidelines in December 2020, which included comprehensive information about deaths among confirmed patients, instructions for healthcare workers’ behaviors, and care for people with symptoms [4]. These guidelines were vital for the disease’s accurate evaluation, diagnosis, treatment, and rehabilitation in terms of finding evidence for organ pathology and injury [5].

Recent studies have shown that infection-control guidelines help individuals prevent, prepare for, respond to, and recover from an infectious disease. Mossa-Basha et al. [6] argued that infection-control guidelines play a critical role in caring for, treating, and handling patients in addition to managing a rapidly increasing number of patients in the healthcare community. Such guidelines can help with resource allocation, coordination, and communication in healthcare settings amid the pandemic [6]. Carlucci et al. [7] reported that failure to comply with the guidelines tends to increase the risk of mortality and of the infection spreading to family, friends, and colleagues. In addition, they suggested that guidelines should be prepared with the use of reliable data to prevent the spread of the disease and create a sense of empathy and urgency among the public. According to Park et al. [8], establishing and distributing COVID-19 infection-control guidelines to the public prevents critical social support and resources from being wasted and minimizes the spread of the disease. In addition, the timely distribution of guidelines would be more effective in preventing a crisis, especially among groups that are vulnerable to diseases.

Public empathy with guidelines is important. Wolff et al. [9] found that a poorer understanding of the guidelines or a lower level of self-control undermined or interfered with compliance with the guidelines.

Mehanna et al. [10] argued that while COVID-19 infection-control guidelines can slow and prevent disease spread and transmission, the deliberate or accidental disregard of guidelines by some individuals can lead to the disease spreading rapidly.

Therefore, this study was focused on perceived severity, perceived susceptibility, and COVID-19 infection-prevention attitude as factors influencing public response to the pandemic and was intended to examine the structure (causality) in which each factor affects infection-prevention behavior. In addition, in this causality, we tried to verify the effect of public empathy for the guidelines for responding to infectious diseases.

Related studies have mostly been conducted on the attitudes and behaviors of infectious disease workers toward infectious diseases such as MERS, SARS, and Ebola [11,12,13,14,15,16,17,18,19,20], as well as studies on the attitudes and perceptions of students in infectious disease-related departments [21,22]. However, this study is different in that it involves the general public, who are subject to infectious diseases, and it is meaningful in that it suggests the direction of crisis management policies by understanding public perceptions and behaviors in responding to infectious diseases.

## 2. Literature Review and Hypothesis Development

### 2.1. Types of COVID-19 Damage

In general, damage due to a disaster is categorized into physical and economic damage. Lee et al. [23], who classified types of disaster damage, divided problems experienced by disaster victims into four categories: property/real estate, workplace, physical, and psychological problems. Lee et al. [24] and Lee and Kim [25] classified disaster damage into physical damage to individuals, including post-traumatic stress, and economic damage to households. 

According to the World Health Organization [26] the most common symptoms of COVID-19 are fever, dry cough, and fatigue. Other less common symptoms that may affect some patients include a loss of taste or smell, nasal congestion, conjunctivitis (also known as red eyes), sore throat, headache, muscle or joint pain, different types of skin rash, nausea or vomiting, diarrhea, and chills or dizziness. Symptoms of severe COVID-19 disease include shortness of breath, loss of appetite, confusion, persistent pain or pressure in the chest, and a high temperature (above 38°C). Others include irritability, confusion, reduced consciousness (sometimes associated with seizures), anxiety, depression, sleep disorders, and more severe and rare neurological complications such as strokes, brain inflammation, delirium, and nerve damage. In addition, Lopez et al. [27], who studied the psychological effects of COVID-19, found that lockdowns caused by COVID-19 contributed to psychological pain and cognitive discomfort in adults. Furthermore, the World Health Organization [28] reported that, as of 27 August 2021, the number of deaths due to COVID-19 worldwide has reached 4,459,381. South Korea is experiencing increasing economic damage caused by the COVID-19 pandemic, with the unemployed and economically inactive population on the rise [29] and consumption falling by 5%, which disproportionately impacts vulnerable groups [30]. Kim and Kim [31] studied the relationship between the number of micro-enterprise stores in Korea and that of COVID-19 patients, and they found a negative correlation between the two variables. In other words, the increasing number of COVID-19 patients affects the continuity of micro-enterprises. Thus, there are increasing economic damage due to rising COVID-19 infections.

### 2.2. Factors Influencing COVID-19 Infection-Prevention Behaviors

#### 2.2.1. Perceived Susceptibility, Perceived Severity, and Behaviors

The health belief model [32,33,34] serves as a critical framework for studying public health behaviors. According to this model, factors affecting public health behaviors include perceived susceptibility, perceived severity, self-efficacy for health behaviors, perceived benefits, perceived barriers, and health motivation. In particular, perceived susceptibility refers to the susceptibility of an individual based on their perception about being exposed to and potentially contracting a disease [35]. Meanwhile, perceived severity is defined as an individual’s perception about facing negative consequences and risks caused by a disease [36]. 

Previous studies have demonstrated that perceived susceptibility and perceived severity precede and affect public health behaviors. Ajzen and Fishbein [37] studied public social behaviors and reported that perceived susceptibility and perceived severity play important roles in predicting potential prevention behaviors. Protection motivation theory [38,39], the extended the parallel process model [40,41], and the risk perception attitude framework [42] divide perceived risk into perceived susceptibility and perceived severity. Rimal and Juon [43] studied the relationship between risk and health-seeking behavior based on their theories and concluded that perceived risk had a positive effect on health-information-seeking behavior.

Iachini et al. [44], who studied social distance perception and behavior in the COVID-19 pandemic, found that regulation of interpersonal space is not an actual objective risk but is influenced by people’s subjective risk perception and related anxiety levels. They also stated that such subjective risk perceptions induce avoidance behavior. Kim and Kim [45] also studied the public’s perceived severity and perceived susceptibility to COVID-19. The results of their study revealed that these two factors are important variables affecting the public’s COVID-19 infection-prevention behaviors. Liau and Zimet [46] reported that a higher level of perceived risk about novel swine-origin influenza virus A led to a higher level of vaccination intention. Xu et al. [47] mentioned that perceived risk is an important variable to predict infection-prevention behaviors such as hand washing, coughing into elbows, wearing masks, and social distancing. In addition, Jo et al. [48] and Bin [49], who examined infection-prevention behaviors in South Korea, noted that perceived severity and perceived susceptibility to tuberculosis had positive effects on infection-prevention behavioral intention. Further, Ji and Moon [50] stated that perceived anxiety about potential disaster damage had a positive effect on evacuation behaviors during a disaster. In addition, Rogers [39], Floyd et al. [51], Witte and Allen [52], and Brug et al. [53] reported that perceived susceptibility and perceived severity are significantly correlated to intentions and behaviors to protect oneself.

In summary, based on the damage caused by the COVID-19 pandemic, the perceived negative consequences of COVID-19 can be divided into perceived severity of physical and economic damage. Furthermore, perceived susceptibility to and perceived severity of COVID-19 have positive effects on COVID-19 infection-prevention behaviors. In view of this, the authors of the current study established the following hypotheses:

Hypothesis 1 (H1).
*Perceived susceptibility to COVID-19 has a positive effect on COVID-19 infection-prevention behaviors.*

Hypothesis 2 (H2).
*Perceived severity of physical damage from COVID-19 has a positive effect on COVID-19 infection-prevention behaviors.*

Hypothesis 3 (H3).
*Perceived severity of economic damage from COVID-19 has a positive effect on COVID-19 infection-prevention behaviors.*

#### 2.2.2. Perceived Susceptibility, Perceived Severity, and Attitudes

Based on the work of Rimal and Real [42] and Witte [40,41], who viewed perceived susceptibility and perceived severity as risk factors, the authors of this study reviewed the following relationship between perceived risk and attitude: Ajzen [54] and Quintal et al. [55] stated that perceived risk determines attitudes and influences behavioral intentions via attitudes. Burns and Slovic [56] revealed that the public’s perceived risk during disease control and prevention is related to their prevention attitudes and behaviors. Bae and Chang [57] reported that affective risk perception for COVID-19 mediates attitudes and positively affects behavioral intention. Yang [58] also demonstrated the relationship between perception and attitude toward an infectious disease by finding a positive correlation between nurses’ perception about tuberculosis infection and tuberculosis infection management attitudes. Kim [59] revealed that perceived risk positively influenced safety attitudes as the public exhibited more proactive attitudes toward safety when they were more aware of or interested in risk factors. Accordingly, we hypothesized that the public’s perceived risk for COVID-19 would have a positive effect on infection-prevention attitudes, as presented with the following hypotheses:

Hypothesis 4 (H4).
*Perceived susceptibility to COVID-19 has a positive effect on COVID-19 infection-prevention attitudes.*

Hypothesis 5 (H5).
*Perceived severity of physical damage from COVID-19 has a positive effect on COVID-19 infection-prevention attitudes.*

Hypothesis 6 (H6).
*Perceived severity of economic damage from COVID-19 has a positive effect on COVID-19 infection-prevention attitudes.*

#### 2.2.3. Attitude and Behavior

Attitudes refer to consistent beliefs in the consequences of particular behaviors [60], with individuals’ attitudes and behaviors forming a hierarchical relationship [61]. The theory of reasoned action [62] and the theory of planned behavior [60,63] postulate that public attitude affects behavioral intention. Jo and Yoo [64] investigated the relationship between the South Korean public’s attitude toward risk and behavioral intention. They reported that people’s attitude toward risk had a positive effect on their risk-prevention behavioral intention. Kang and Yang [65] found that people’s attitude toward tuberculosis prevention positively influenced their tuberculosis-prevention behavioral intention. 

Studies have recently been published on the correlation between the public’s infection-prevention attitude and behavior during COVID-19. The public attitude toward COVID-19 is defined as the belief or confidence of overcoming COVID-19 through civilized and responsible behaviors when COVID-19 symptoms appear [66]. Chan et al. [67] found a statistically significant difference between public attitude and actual practice to prevent infections during the COVID-19 pandemic. Erfani et al. [68] revealed a positive correlation between public attitude and behavior toward COVID-19 infection prevention. According to Puspitasari et al. [69], positive attitude and behavior toward COVID-19 have controlled the spread of the disease. Similarly, Xu et al. [47] stated that attitude toward COVID-19 is a strong variable that can be used in predicting infection-prevention behaviors. In sum, the abovementioned studies and theories suggest that public attitude toward COVID-19 infection prevention can positively influence infection-prevention behavior. Accordingly, the authors of this study established the following hypothesis:

Hypothesis 7 (H7).
*The public’s infection-prevention attitude toward COVID-19 has a positive effect on infection-prevention behavior.*

### 2.3. Social Empathy

Policy awareness is a key factor determining participation [70]. A government’s unilateral or top–down policy implementation can lead to a policy failure or deadlock [71]. In this context, researchers highlight why citizen participation is necessary for the justification and acceptance of government policies among citizens [72]. Citizen science responds to calls of the day to seek solutions about problems in everyday life beyond efforts to address science and technology problems in terms of improving scientific competencies and national competitiveness [72]. In other words, the public perception of and empathy with regulations or guidelines have profound effects on the acceptance of those regulations. Putnam [73] stated that social empathy can encourage social participation from members of society and create social policies and programs. Social empathy is part of social capital and includes social organizational characteristics such as networks, norms, and social trust to promote coordination and cooperation for the benefit of members of society [74]. In particular, Dryhurst et al. [75] found that public support for COVID-19 safety and health guidelines had a significant effect on the spread of the disease. Considering these studies, social empathy may moderate people’s perception, attitude, and behavior. Hence, the authors of this study established the following hypotheses:

Hypothesis 8 (H8).
*Public empathy with COVID-19 infection-control guidelines moderates the effect of the perceived susceptibility to COVID-19 on infection-prevention behaviors.*

Hypothesis 9 (H9).
*Public empathy with COVID-19 infection-control guidelines moderates the effect of the perceived severity of physical damage from COVID-19 on infection-prevention behaviors.*

Hypothesis 10 (H10).
*Public empathy with COVID-19 infection-control guidelines moderates the effect of the perceived severity of economic damage from COVID-19 on infection-prevention behaviors.*

Hypothesis 11 (H11).
*Public empathy with COVID-19 infection-control guidelines moderates the effect of the perceived susceptibility to COVID-19 on infection-prevention attitudes.*

Hypothesis 12 (H12).
*Public empathy with COVID-19 infection-control guidelines moderates the effect of the perceived severity of physical damage from COVID-19 on infection-prevention attitudes.*

Hypothesis 13 (H13).
*Public empathy with COVID-19 infection-control guidelines moderates the effect of the perceived severity of economic damage from COVID-19 on infection-prevention attitudes.*

Hypothesis 14 (H14).
*Public empathy with COVID-19 infection-control guidelines moderates the effect of infection-prevention attitudes on infection- prevention behaviors.*

### 2.4. Conceptual Model

**Figure 1:**
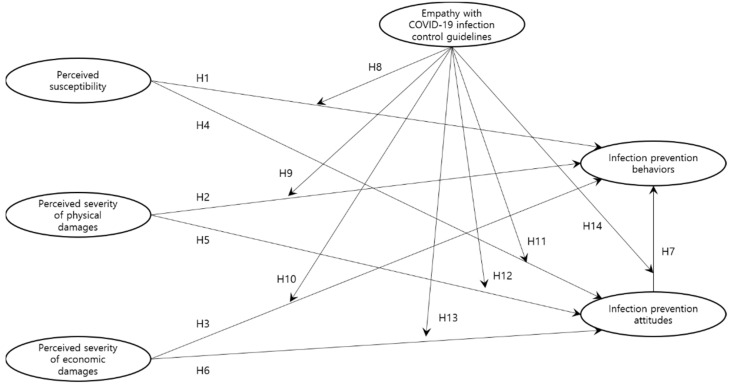
Conceptual Model of The Study.

Considering the previously discussed 11 hypotheses regarding the relationships between the public’s perceived severity, perceived susceptibility, COVID-19 infection-prevention attitude, and infection-prevention behavior, as well as the moderating effects of public empathy with COVID-19 infection-control guidelines, the authors of this study established a conceptual model, as illustrated in Figure 1.

## 3. Materials and Methods

### 3.1. Data Collection

Data were collected with self-administered questionnaires that were distributed by Macromill Embrain, an online marketing research firm, via e-mail to a panel of adults 18 years or older for six days from 11 to 19 October, 2020. Online questionnaires were randomly distributed to the panel from the company, and proportional sampling was conducted considering the population’s gender, age, and place of residence. The study was conducted with the aim of collecting more than 200 cases as effective samples with reference to Park et al. [76], Jong et al. [77], and Rezaei et al. [78], who used the survey data of adult Korean men and women. A total of 211 questionnaire copies were collected, resulting in a confidence level of 95 ± 7% of sample error when for population of adult men and women between the ages of 20 and 70 in South Korea. It was confirmed that there were no missing responses, outliers, and duplicate responses in the collected data. Subsequently, these data were used for analysis.

### 3.2. Measures

Measures were developed to examine perceived susceptibility, perceived severity of physical damage, perceived severity of economic damage, COVID-19 infection-prevention attitude, COVID-19 infection-prevention behavior, and public empathy with COVID-19 infection-control guidelines, as presented in Table 1.

#### 3.2.1. Perceived Susceptibility

Rosenstock [35] defined perceived susceptibility as the level of susceptibility perceived by an individual about contracting a disease. Bae and Chang [57] measured perceived risk in terms of people’s concern about contracting COVID-19 for themselves and their family members. Accordingly, the authors of the current study measured this variable as the possibility of contracting COVID-19. All variables were measured on a 5-point Likert scale (1 point = strongly disagree; 5 points = strongly agree).

#### 3.2.2. Perceived Severity

Perceived severity was measured by the level of severity that an individual perceives for physical and economic damage based on Becker’s definition [36] and studies on types of damage from COVID-19 [23,24,25,26,79]. All variables were measured on a 5-point Likert scale (1 point = strongly disagree; 5 points = strongly agree).

#### 3.2.3. COVID-19 Infection-Prevention Attitudes

Kumar et al. [66] and Chan et al. [67] measured infection-prevention attitudes toward COVID-19 as the confidence in overcoming COVID-19, as well as the belief in and social responsibility toward complying with COVID-19 guidelines and sharing useful information. Therefore, the authors of this study established questions regarding COVID-19 infection-prevention attitude to prevent the spread of the virus. All variables were measured on a 5-point Likert scale (1 point = strongly disagree; 5 points = strongly agree).

#### 3.2.4. COVID-19 Infection-Prevention Behaviors

The Central Disaster Management Headquarters in South Korea [80] stated that wearing masks, frequently washing hands, and avoiding contact with others through social distancing can effectively prevent COVID-19 infections. Lee and You [81], Galea et al. [82], Smith et al. [83], and Song and Yoo [84] noted the role of these behavioral traits in the prevention of COVID-19 infection. Furthermore, in the measurement of COVID-19 infection-prevention behavior with four questions, Yoo and Song [85] included avoiding going outside and stay-at-home protocols and Xu et al. [47] included adopting correct coughing habits. To measure COVID-19 infection-prevention behavior, the authors of this study used three common behaviors observed in previous studies: wearing masks, frequently washing hands, and social distancing. All variables were measured on a 5-point Likert scale (1 point = strongly disagree; 5 points = strongly agree).

#### 3.2.5. Empathy with COVID-19 Infection-Control Guidelines

The Central Disaster Management Headquarters in South Korea [80] prepared and implemented guidelines on stricter hygiene and social distancing to stop the spread of COVID-19. The guidelines made it mandatory to wear masks in public places and strengthened social distancing. In addition, to prevent the spread of the virus due to public gatherings, the opening of schools was delayed, people living in close contact with COVID-19 patients were required to take a COVID-19 test, and COVID-19-related information )including contact tracing for COVID-19-confirmed patients) was shared. Hence, the authors of this study measured public empathy with the four abovementioned government guidelines to prevent the spread of COVID-19. All variables were measured on a 5-point Likert scale (1 point = strongly disagree; 5 points = strongly agree).

#### 3.2.6. Statistical Analysis

All statistical analyses were conducted using SPSS software version 26.0 and SPSS AMOS software version 22.0 (IBM SPSS Inc., Chicago, IL, USA). The demographic characteristics of the respondents were examined using frequency analysis. Descriptive statistical analysis was used for the baseline values of the measures, such as the mean and standard deviation. Confirmatory factor analysis (CFA) and reliability analysis were conducted to evaluate the reliability of the measures. The model fit for CFA results was determined based on the work of Joreskog and Sorbom [86], Byrne [87], and Tobbin [88]. In addition, standardized estimates and variance estimates from the CFA analysis were used to calculate the composite reliability (CR), average variance extracted (AVE), and square roots of the AVEs; Cronbach’s α from the reliability analysis was confirmed. The indices were used to determine the reliability of the measures based on reference values from Fornell and Larcker [89], Nunnally [90], and Chen et al. [91]. Structural equation modeling (SEM) was used to test Hypotheses 1–7, while Woo’s [92] proposed method of testing the moderating effect was used to test Hypotheses 8–13. First, k-means cluster analysis was conducted by inputting all variables that constitute public empathy with COVID-19 infection-control guidelines, and the number of groups was input as 2, dividing the respondents into a group with high empathy and a group with low empathy. In addition, an independent *t*-test was conducted to review the difference in the average of latent variables for each group. A multi-group SEM was then conducted, and the difference between path coefficients in each group was determined in terms of the critical ratio (CR) for differences between parameters in pairwise parameter comparisons. An absolute critical ratio of ≥1.965 indicates a statistically significant difference in the path coefficient, and the presence of a moderating effect can be determined [92]. 

## 4. Results

### 4.1. Sample

A total of 211 responses were analyzed. Table 2 lists the demographic characteristics of the respondents. Of the respondents, approximately 50.7% were male and 49.3% were female. Regarding their age, approximately 24.6%, 25.6%, 25.6%, 25.6%, and 24.2% were in their 20s, 30s, 40s, and 50s and older, respectively. In terms of their place of residence, approximately 64.0% of the respondents lived in the Seoul metropolitan area, 10.9% lived in the Chungcheong and Gangwon region, 19.9% lived in the Gyeongsang region, and 5.2% lived in the Jeolla region. Regarding their educational background, approximately 14.7% were high school graduates or had a lower educational background, 13.3% were junior college graduates, 60.2% were four-year university graduates, and 11.8% were graduate school graduates. In terms of their type of employment, approximately 52.6% were regular workers, 6.2% were part-time workers, 2.8% were freelancers, and 38.4% belonged to other types. Regarding their average monthly household income, those with ≥5 million won accounted for the highest percentage with approximately 33.2%, followed by those with ≥2 million won, and those with <3 million won.

### 4.2. Measurement Model

Content, convergent, and discriminant validity were evaluated to determine whether the measures were fit. For content validity, the measures constituted a study model based on the literature. For convergent validity, indices from CFA were referenced. The results showed that the chi-square distribution (χ 2/df) was 1.872, the root mean square residual (RMR) was 0.033, the root mean square error of approximation (RMSEA) was 0.064, the goodness of fit index (GFI) was 0.927, the adjusted GFI (AGFI) was 0.902, the normed fit index (NFI) was 0.921, the Tucker–Lewis index (TLI) was 0.970, and the comparative fit index (CFI) was 0.956. The standardized estimate, CR, AVE, and Cronbach’s α were confirmed. The standardized estimate was above 0.6, and the CR and AVE values for the constructs were above 0.9. Cronbach’s α was above 0.7 for all constructs (Table 3). The indices support their fit based on the work of Fornell and Larcker [89]. Hence, convergent validity for the measures was considered adequate in this study. Finally, to evaluate discriminant validity, the inter-construct correlation coefficients and the square roots of the AVEs were compared. The results showed that the latter were higher than the former, demonstrating discriminant validity [90]. The square roots of the AVE are italicized and underlined.

### 4.3. Structural Model 1

**Figure 2:**
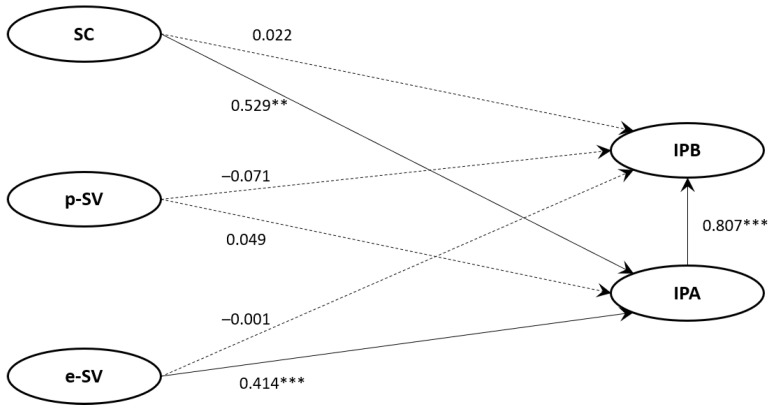
Structural Model with The Standardized Path Estimate. Note: ** *p* < 0.01, *** *p* < 0.001. Abbreviations: SC, perceived susceptibility; p-SV, perceived severity of physical damage; e-SV, perceived severity of economic damage; IPA, infection-prevention attitude; IPB, infection-prevention behavior.

The fit indices in the structural model were χ2/df = 1.831, RMR = 0.056, RMSEA = 0.062, GFI = 0.938, AGFI = 0.906, TLI = 0.959, and CFI = 0.970. The model was accordingly considered acceptable. First, perceived susceptibility (SC) was found to positively affect infection-prevention attitudes (IPA; β = 0.529, *p* < 0.01), supporting H4. Second, perceived severity of economic damage (e-SV) had a positive effect on infection-prevention attitude (IPA; β = 0.414, *p* < 0.001), supporting H6. Third, infection-prevention attitude (IPA) had a positive effect on infection-prevention behavior (IPB; β = 0.807, *p* < 0.001), supporting H7. Meanwhile, the effect of the perceived severity of physical damage (p-SV) on infection-prevention attitude (IPA; H2), the direct effects of perceived susceptibility (SC), the perceived severity of physical damage (p-SV), and the perceived severity of economic damage (e-SV) on infection-prevention behavior (H4–6) did not have statistically significant effects; therefore, the relevant hypotheses were rejected (Figure 2). 

### 4.4. Groups Categorized by the Public’s Empathy with COVID-19 Infection-Control Guidelines

To verify the moderating effect of the respondents’ empathy with COVID-19 infection-control guidelines, they were divided into two groups: one with high empathy and one with low empathy. The authors of this study examined whether there were any differences in each group’s perceived susceptibility, perceived severity of physical damage, perceived severity of economic damage, infection-prevention attitudes, and infection-prevention behaviors. The results showed that there was no difference in the perceived susceptibility and perceived severity of physical damage between the groups with high versus low empathy (Table 4).

### 4.5. Structural Model 2

**Figure 3:**
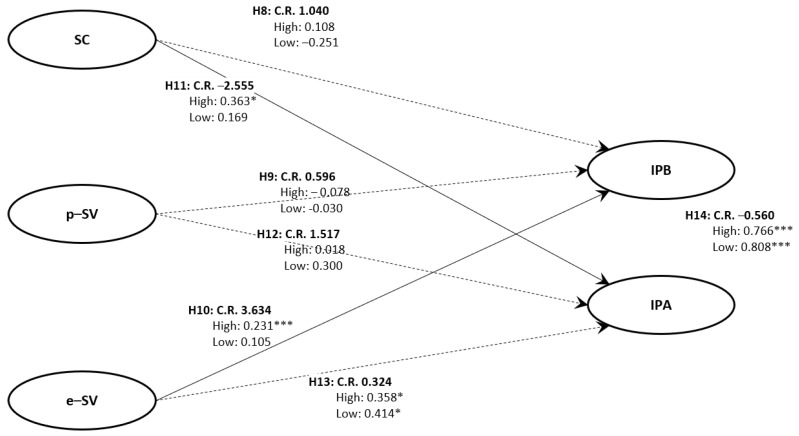
Structural Model Comparing The Standardized Path Estimate and Composite Reliability in the High and Low Informational Support Groups. Note: * *p* < 0.05, *** *p* < 0.001. Abbreviations: CR, critical ratio; SC, perceived susceptibility; p-SV, perceived severity of physical damage; e-SV, perceived severity of economic damage; IPA, infection-prevention attitude; IPB, infection-prevention behavior.

Multi-group SEM was conducted to confirm the moderating effect of empathy with COVID-19 infection-control guidelines. The model fit was χ2/df = 1.342, RMR = 0.066, RMSEA = 0.040, GFI = 0.912, AGFI = 0.893, TLI = 0.960, and CFI = 0.971; thus, the model was considered acceptable. The results showed that the respondents’ empathy with COVID-19 infection-control guidelines moderated the effect of the perceived severity of economic damage on infection-prevention behaviors (CR = 3.634), supporting H10. In the group with high empathy with COVID-19 infection-control guidelines, the perceived severity of economic damage had a statistically significant effect on infection-prevention behaviors (β = 0.231, p < 0.001), unlike in the group with low empathy. Next, the perceived severity of economic damage was found to moderate the effect of perceived susceptibility on infection-prevention attitude (CR = −2.555), supporting H11. In the group with high empathy with COVID-19 infection-control guidelines, perceived susceptibility had a statistically significant effect on infection-prevention behavior (β = 0.363, p < 0.05), unlike in the group with low empathy. The absolute CR for H8, H9, H12, H13, and H14 did not exceed 1.965; therefore, these hypotheses were rejected (Figure 3).

## 5. Discussion

The purpose of this study was to verify whether public empathy with COVID-19 infection-control guidelines moderates the causal relationship between perceived susceptibility, perceived severity, infection-prevention behavior, and infection-prevention attitude. It allowed us to explore a method of effectively preventing the spread of infections at a time when an infectious disease is rapidly spreading. 

The results of the study are as follows. First, the perceived susceptibility and perceived severity of economic damage had positive effects on infection-prevention attitude, and infection-prevention attitude had a positive effect on infection-prevention behavior. In other words, the perceived susceptibility and perceived severity of economic damage affect infection-prevention behavior via infection-prevention attitude. These results may suggest that the more the public tends to perceive that they will contract an infectious disease and suffer economic damage, the higher their infection-prevention attitudes and the more proactive they are in adopting infection-prevention behaviors. These results are supported by the work of Burn and Slovic [56], who found that perceived risk influenced prevention attitudes and behaviors during disease prevention and control, as well as by the work of Bae and Chang [57], who reported that perceived risk affected behavior via attitude. 

Second, the perceived severity of economic damage had a positive effect on infection-prevention attitude. Meanwhile, the perceived severity of physical damage had no significant effect on infection-prevention attitude. These results are supported by the work of Jang [30], who reported severe economic damage to vulnerable groups and a phenomenon in South Korea where, although the social distancing used to counter COVID-19 spread limited the economic activities of self-employed people, they still complied with infection-control guidelines to facilitate returning to their normal lives sooner. In addition, companies have prepared infection-control measures in cooperation with local governments, with some complaining about not following stricter infection-control protocols for some facilities [93,94]. As of September 2021, Seoul has limited the number of people allowed for a gathering to six, and no violations have yet been detected [95]. 

Third, public empathy with infection-control guidelines was found to positively moderate the effect of the perceived severity of economic damage on infection-prevention behavior and that of perceived susceptibility on infection-prevention attitude. Given this study’s results showing that infection-prevention attitude has a positive effect on infection-prevention behavior, public empathy with infection-control guidelines is considered to be effective in encouraging the public to perform more actively infection-prevention behaviors. These results are supported by the work of Dryhurst et al. [75], who argued that public support for COVID-19 safety and health guidelines influenced the spread of the disease, and Putnam [73], who demonstrated the positive effect of social empathy. 

## 6. Conclusions

Based on our results, the level of public infection-prevention behaviors can be increased amid the spread of an infectious disease in the following ways. First, it is important to engage in risk communication regarding a potential viral infection and cooperation to prevent any further spread. The perception that the spread of a virus should be swiftly prevented by seriously considering the susceptibility to the virus and potential economic damage leads to more proactive infection-prevention behaviors by the public. Second, multifaceted efforts are required to increase public empathy with infection-control guidelines to prevent the spread of an infectious disease. It is necessary to request compliance with infection-control guidelines after disseminating them, promoting them, and explaining why they are necessary in a way that is comprehensible to the public. Third, the public needs to pay attention to the process of preparing policies related to the pandemic to prevent the spread of infectious disease and make suggestions to doctors and quarantine authorities. In order to increase the public consensus on the pandemic policies to prevent the spread of infectious diseases, as in the second proposal, special efforts by quarantine authorities are necessary. However, it is also important for the public to be well aware of the pandemic policy preparation process and to reasonably revise these policies through sufficient communication on points of contention and suggested improvements. Forth, measures should be implemented to alleviate or reduce economic damage to the public in a viral pandemic. While viral infections directly cause physical damage, the results of this study suggest that the public is more sensitive to perceived economic damage than perceived physical damage. This is because perceived physical damage does not affect infection-prevention behavior but perceived economic damage has a positive effect on infection-prevention behavior via infection-prevention attitude.

## 7. Strengths and Limitations

This study is significant in that it has verified the effect of public sympathy in examining the government’s response to infectious diseases. Many studies related to infectious disease management and its response have focused on the attitudes and perceptions of medical professionals rather than public awareness and sympathy [96,97,98]. In contrast, this study has verified the importance of public awareness and sympathy in future infectious disease management response by confirming the effect of public awareness and sympathy on infectious disease response behavior.

This study had the following limitations. First, are the spatial and temporal limitations. Spatially, the study was only conducted on adults residing in South Korea, and temporally, the study was conducted at one point in 2020. Similar studies should be conducted in multiple countries. In addition, a time-series survey should be conducted to compare and analyze changes in public perception and sympathy according to the spread of infectious diseases and changes in response policies. Second, this study did not use demographic characteristics such as gender and age as variables. Such variables should be included in future studies. Third, this study used perceived susceptibility, perceived severity, infection-prevention attitude, and public empathy with infection-control guidelines to predict infection-prevention behavior. However, the public’s infection-prevention behavior may be affected by other variables such as an individual’s environment or information literacy about infectious diseases. Accordingly, more variables need to be comprehensively considered in the future. 

## Figures and Tables

**Table 1:** Constructs and Survey Questionnaire.

Construct 1: Perceived susceptibility (SC) SC 1: I think it might be possible that I get infected with COVID-19. SC 2: I think it might be possible that my family members get infected with COVID-19.
Construct 2: Perceived severity of physical damage (p-SV) p-SV 1: I think the spread of COVID-19 increases the extent of physical damage. p-SV 2: I think the development of COVID-19 increases the extent of physical damage.
Construct 3: Perceived severity of economic damage (e-SV) e-SV 1: I think COVID-19 has a negative impact on my local economy. e-SV 2: I think COVID-19 has a negative impact on other local economies.
Construct 4: COVID-19 infection-prevention attitude (IPA) IPA 1: I think I should follow infection-control guidelines despite inconveniences. IPA 2: I think I should follow infection-control guidelines despite personal costs. IPA 3: I think I should follow infection-control guidelines with a sense of social responsibility.
Construct 5: COVID-19 infection-prevention behavior (IPB) IPB 1: I am practicing social distancing to prevent COVID-19 infection. IPB 2: I always wear a mask to prevent COVID-19 infection. IPB 3: I frequently wash my hands to prevent COVID-19 infection.
Construct 6: Public empathy with COVID-19 infection-control guidelines (RE) RE 1: I empathize (agree) with social distancing guidelines to prevent COVID-19 infections. RE 2: I empathize (agree) with masking guidelines to prevent COVID-19 infections. RE 3: I empathize (agree) with delaying the opening of schools to prevent COVID-19 infections. RE 4: I empathize (agree) with requiring COVID-19 tests to prevent COVID-19 infections. RE 5: I empathize (agree) with sharing information to prevent COVID-19 infections.

**Table 2:** Demographic Characteristics of the Sample.

Characteristics		Frequency	Percentage
Gender	Male	107	50.7
	Female	104	49.3
Age	20s	52	24.6
	30s	54	25.6
	40s	54	25.6
	50s and older	51	24.2
Place of residence	Seoul metropolitan area	135	64.0
	Chungcheong and Gangwon region	23	10.9
	Gyeongsang region	42	19.9
	Jeolla region	11	5.2
Educational background	High school graduation or lower	31	14.7
	Junior college graduation	28	13.3
	4-year university graduation	127	60.2
	Graduate school graduation	25	11.8
Type of employment	Regular worker	111	52.6
	Part-time worker	13	6.2
	Freelancer	6	2.8
	Housewife, student, and others	81	38.4
Average monthly household income *	<2 million won	22	10.5
	≥2 million won to <3 million won	43	20.4
	≥3 million won to <4 million won	37	17.5
	≥4 million won to <5 million won	39	18.5
	≥5 million won	70	33.2

Note: * 10,000 South Korean won (USD 1 = KRW 1155.9).

**Table 3:** Results of Reliability Analysis for The Measures.

Construct	Measures	Standardized Estimate	Cronbach’s α	CR	AVE	Inter-Construct Correlations						Mean (SD)
						SC	p-SV	e-SV	IPA	IPB	PE	
Perceived susceptibility	SC1	0.736	0.942	0.947	0.900	0.949						3.730 (0.569)
	SC2	0.622										
Perceived severity of physical damage	p-SV1	0.959	0.856	0.999	0.998	0.392	0.999					2.936 (0.763)
	p-SV2	0.780										
Perceived severity of economic damage	e-SV1	0.877	0.778	0.987	0.975	0.505	0.046	0.987				3.780 (0.793)
	e-SV2	0.689										
Infection-prevention attitude	IPA1	0.893	0.929	0.999	0.996	0.503	0.131	0.527	0.998			4.321 (0.701)
	IPA2	0.912										
	IPA3	0.905										
Infection-prevention behavior	IPB1	0.791	0.814	0.978	0.939	0.42	0.055	0.419	0.820	0.969		4.409 (0.594)
	IPB2	0.909										
	IPB3	0.852										
Infection-control guidelines Public empathy	RE1	0.840	0.788	0.998	0.988	0.369	0.041	0.509	0.696	0.655	0.994	4.002 (0.576)
	RE2	0.773										
	RE3	0.696										
	RE4	0.744										
	RE5	0.612										

Abbreviations: CR, composite reliability; AVE, average variance extracted; SC, perceived susceptibility; p-SV, perceived severity of physical damage; e-SV, perceived severity of economic damage; IPA, infection-prevention attitude; IPB, infection-prevention behavior; PE, infection-control guidelines public empathy; SD, standard deviation; underlined numbers: square roots of AVE.

**Table 4:** Differences in the Means for Constructs according to the Public’s Empathy with COVID-19 Infection-control Guidelines.

Construct	Mean	Standard Deviation	Empathy Group	Mean	Standard Deviation	T-Value
Perceived susceptibility	3.730	0.569	High	3.781	0.581	1.961
			Low	3.615	0.529	
Perceived susceptibility Perceived severity of physical damage	2.936	0.763	High	2.908	0.816	−0.894
			Low	3.000	0.631	
Perceived severity of economic damage	3.780	0.793	High	3.908	0.759	3.612 ***
			Low	3.492	0.798	
Infection-prevention attitudes	4.321	0.701	High	4.505	0.550	5.347 ***
			Low	3.908	0.822	
Infection-prevention behaviors	4.409	0.594	High	4.564	0.479	5.396 ***
			Low	4.062	0.679	

Note: *** *p* < 0.001.

## Data Availability

Not applicable.
